# Genetic landscape of pediatric seizures in Southeast China: identification of a novel GLI3 frameshift variant through whole-exome sequencing

**DOI:** 10.1097/MS9.0000000000005448

**Published:** 2026-08-06

**Authors:** Shan Hong, Dandan Han, Ming Qi, Shan Huang, Min Ren, Linghui Ding, Xiuhong Huang, Wenliu Qiu, Jiang Song, Yanqian Huang, Xiuli Li, Huanming Chen, Xuelei Wang, Liangneng Zou, Yanli Wei

**Affiliations:** a Department of Pediatrics, The Fifth Hospital of Xiamen, Xiamen, Fujian, China; b Department of General Practice, Maxiang Street Community Health Service Center, Xiang’an District, Xiamen, Fujian, China; c Department of General Practice, Guankou Hospital, Jimei District, Xiamen, Fujian, China

**Keywords:** GLI3 frameshift variant, pediatric seizures, Southeast Chinese population, whole-exome sequencing

## Abstract

**Background:**

Pediatric seizure disorders are clinically and genetically heterogeneous. Whole-exome sequencing has improved the detection of rare genetic variants in childhood epilepsy; however, data from pediatric populations in Southeast China remain limited. This study aimed to characterize the genetic landscape of pediatric seizure disorders in Southeast China and to evaluate the clinical diagnostic yield of whole-exome sequencing.

**Materials and methods:**

This retrospective observational study included 21 pediatric patients with seizure disorders who were recruited at the Fifth Hospital of Xiamen, Fujian, China, between January 2021 and June 2024. Clinical data were extracted from medical records. Whole-exome sequencing was performed on DNA extracted from peripheral blood. Sequence variants were annotated, filtered, and classified according to the guidelines of the American College of Medical Genetics and Genomics and the Association for Molecular Pathology. Copy-number variants were evaluated using exome-based algorithms. Descriptive statistics were used because of the limited sample size.

**Results:**

WES identified three clinically relevant, likely pathogenic findings in 3 of 21 patients, corresponding to a provisional diagnostic yield of 14.3%. The remaining 62 of 65 variants were of uncertain significance (VUS). The three retained variants included a GLI3 frameshift variant (exon 2: c.90_91insCAGATGTGAGC; p.Glu31Glnfs*3) and two copy-number variants (16p13.12-16p13.11 duplication and Xp22.31 deletion) with established clinical significance. Functional analysis of all 65 variants revealed that ion channel genes and neurodevelopmental genes were the most frequently affected categories.

**Conclusion:**

Whole-exome sequencing identified clinically relevant genetic findings in a subset of Southeast Chinese children with seizure disorders. The novel GLI3 frameshift variant may suggest an expansion of the GLI3-associated phenotypic spectrum, but further segregation, functional validation, and larger cohort studies are needed. The high proportion of variants of uncertain significance highlights the ongoing challenges of genetic interpretation in pediatric seizure disorders.

## Introduction

Seizure disorders are among the most common neurological conditions in children, affecting approximately 4–10% of children worldwide before the age of 16^[^1^]^. These disorders encompass a spectrum of presentations, including febrile seizures, non-febrile seizures, and mixed types, each with potentially distinct genetic underpinnings^[^2^]^. The impact of these conditions extends beyond the immediate medical concerns, significantly affecting children’s development, educational outcomes, and overall quality of life^[^3^]^.

HIGHLIGHTS

- Whole-exome sequencing was performed on 21 children with seizure disorders from Southeast China.
- Three clinically relevant likely pathogenic findings were identified, corresponding to a provisional diagnostic yield of 14.3%.
- A novel GLI3 frameshift variant was detected in a child with febrile seizures without classical syndromic features.
- Most detected variants were classified as variants of uncertain significance, highlighting the challenges in pediatric seizure genomics.
- These findings support the potential value of genomic testing in underrepresented pediatric populations, while emphasizing the need for larger, validated cohorts.

Recent years have witnessed substantial advances in our understanding of the genetic basis of pediatric seizure disorders. The advent of next-generation sequencing technologies, particularly whole-exome sequencing (WES), has revolutionized the field of medical genetics by enabling comprehensive identification of rare and novel genetic variants^[^4^]^. WES has proven particularly valuable in pediatric neurology, where genetic factors play a crucial role in disease etiology^[^5^]^. Studies have identified numerous genes associated with various forms of epilepsy and seizure disorders, including those encoding ion channels, neurotransmitter receptors, and proteins involved in neural development^[^6^]^. In addition to genetic factors, social determinants of health can shape childhood epilepsy outcomes and access to care^[^7^]^.

However, the genetic landscape of pediatric seizure disorders in Chinese populations remains incompletely characterized. Most previous studies have focused on Western populations, mixed international cohorts, or selected epilepsy syndromes. Population-specific genetic variation may influence diagnostic yield and variant interpretation, particularly in underrepresented populations for which reference data remain limited. Previous studies have suggested potential population-specific genetic variations^[^8,9^]^, highlighting the need for targeted investigations in different ethnic groups. Understanding these genetic patterns is crucial for accurate diagnosis, proper genetic counseling, and the development of personalized treatment strategies^[^10^]^. This study aimed to investigate the genetic basis of seizure disorders in a cohort of pediatric patients from Southeast China using WES. We sought to identify causative variants, characterize their distribution across different types of seizure disorders, and evaluate their potential clinical implications. Additionally, we aimed to compare our findings with those reported in other populations to better understand the genetic heterogeneity of these conditions. The specific objectives were to: (1) identify clinically significant genetic variants; (2) estimate the diagnostic yield of WES in this cohort; and (3) characterize the functional distribution of detected variants.

## Material and methods

### Study design and patient selection

**Figure 1.**
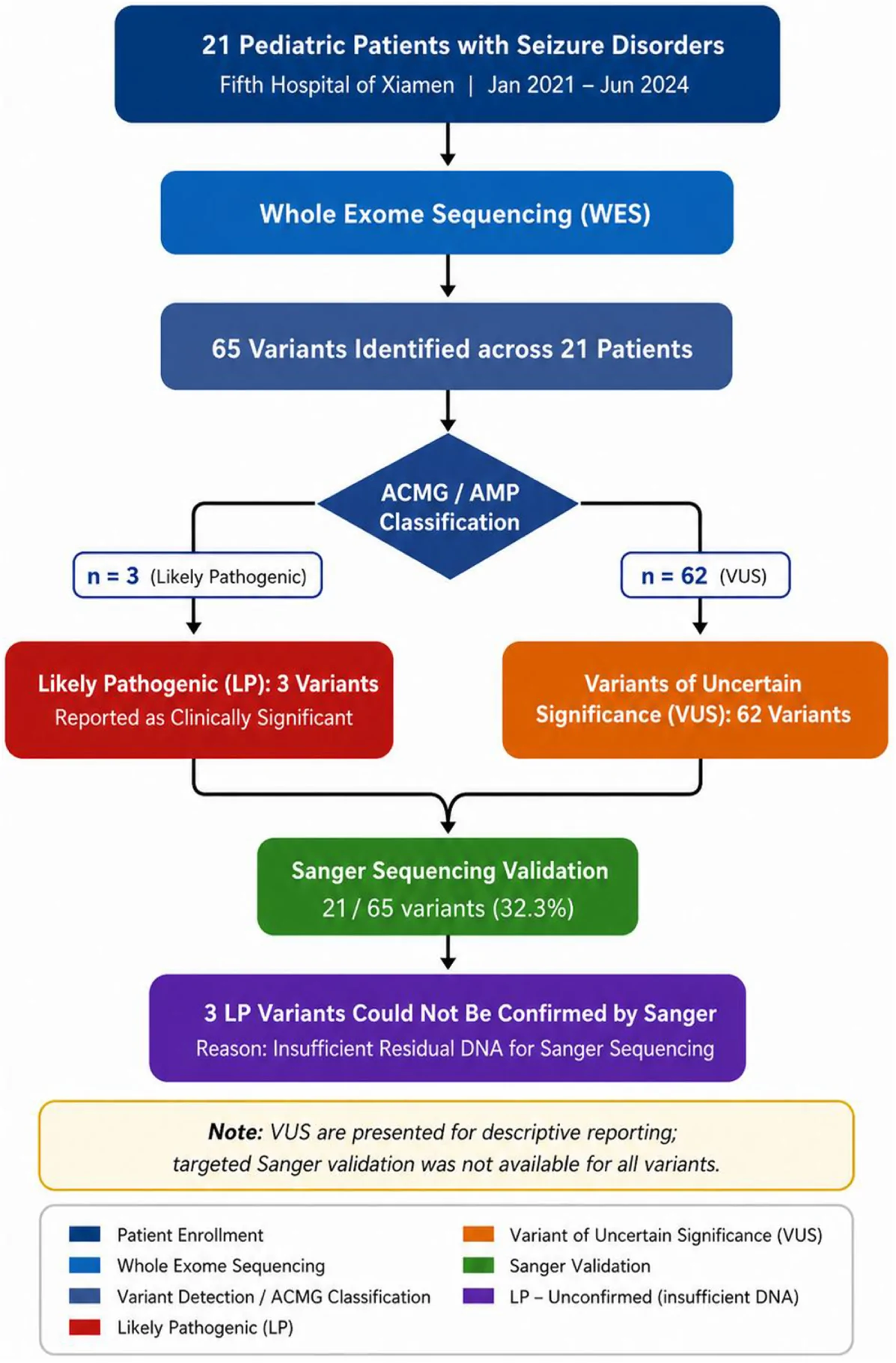
Flowchart of patient selection and variant classification. The diagram illustrates the enrollment of 21 pediatric patients from the Fifth Hospital of Xiamen, the whole-exome sequencing pipeline, variant filtering, ACMG/AMP classification steps, and the final classification outcomes (3 likely pathogenic findings and 62 VUS).

This retrospective observational study included pediatric patients with seizure disorders who were recruited from the Fifth Hospital of Xiamen, Fujian, China, between January 2021 and June 2024. The inclusion criteria were as follows: (1) age ≤10 years; (2) at least one documented seizure episode; and (3) consent from parents or legal guardians for genetic testing. The exclusion criteria were as follows: (1) previously confirmed genetic diagnosis before enrollment; (2) severe organ dysfunction; or (3) insufficient clinical information for analysis. Clinical information was extracted from medical records, including age, sex, seizure type, comorbidities, family history, and available genetic testing results. Because of the retrospective design, clinical documentation varied in completeness. Written informed consent was obtained from all participants’ legal guardians, and the study was approved by the Ethics Committee of the Fifth Hospital of Xiamen (Approval No. XMWY-KY-2024-022-01). The study design is shown in Fig. 1. The study adhered to the Declaration of Helsinki.

### Sample collection and DNA extraction

Peripheral blood samples were collected from each patient, and DNA was extracted using the QIAamp DNA Blood Mini Kit (Qiagen), following the manufacturer’s protocol. DNA concentration and purity were assessed before sequencing.

### Whole-exome sequencing

WES was performed using the Illumina NextSeq 500 platform. Exome capture was achieved using the TruSeq DNA Exome Kit (Illumina), and the libraries were sequenced with 150-bp paired-end reads. The mean sequencing depth was approximately 100×. More than 95% of target exonic regions achieved at least 20× coverage, and more than 90% achieved at least 50× coverage. All samples passed quality-control thresholds.

### Bioinformatics analysis and variant classification

Raw sequencing data underwent comprehensive bioinformatics processing following established best practices. Quality control of raw FASTQ files was performed using FastQC (v0.11.9), and adapter sequences were trimmed using Trimmomatic (v0.39). High-quality reads were aligned to the human reference genome (GRCh37/hg19) using BWA-MEM (v0.7.17) with default parameters optimized for paired-end sequencing data.

Variant calling was conducted using the Genome Analysis Toolkit (GATK) HaplotypeCaller (v4.2.0), following GATK Best Practices guidelines. Base quality score recalibration and variant quality score recalibration were applied to improve variant-calling accuracy. Both single-nucleotide variants (SNVs) and small insertions and deletions (indels) were called simultaneously, with variants required to have a minimum quality score of 30 and a read depth of ≥10×.

Comprehensive variant annotation was performed using ANNOVAR (v2020-06-08), incorporating multiple databases and prediction algorithms. Functional annotation included transcript-based annotation (RefGene), amino acid change prediction, and splice-site impact assessment. Population frequency data were extracted from gnomAD version 3.1.2, with particular emphasis on East Asian population frequencies (AF_eas) given our study population demographics.

Variant filtering and prioritization employed a tiered approach based on inheritance patterns and population frequencies. For variants in genes associated with autosomal dominant conditions, we applied a stringent minor allele frequency threshold of <0.001 in the East Asian population in gnomAD (AF_eas). For presumed autosomal recessive conditions, a more lenient threshold of <0.01 was applied, allowing for potential carrier frequencies in the population. Variants with higher population frequencies were flagged but retained for analysis when supported by strong functional evidence.

Pathogenicity assessment used multiple in silico prediction algorithms, including REVEL (pathogenic threshold ≥ 0.5), SIFT (deleterious prediction), PROVEAN (deleterious threshold ≤ −2.5), and MutationTaster (disease-causing prediction). Clinical significance was evaluated through ClinVar database queries and OMIM phenotype correlations. All variants were systematically classified according to the 2015 guidelines of the American College of Medical Genetics and Genomics (ACMG) and the Association for Molecular Pathology (AMP) ^[^11^]^, incorporating the following evidence types: 1. Very Strong Evidence (PVS1): null variants in genes where loss of function is established as a disease mechanism; 2. Strong Evidence (PS1-PS4): including identical amino acid changes of known pathogenicity and de novo occurrence in affected individuals; 3. Moderate Evidence (PM1-PM6): including location in functional domains, novel missense changes, and co-segregation with disease; and 4. Supporting Evidence (PP1-PP5): including computational prediction support and phenotype specificity. Pathogenicity interpretation was also refined by incorporating gene constraint metrics, including pLI and LOEUF scores from the gnomAD database. For loss-of-function (LoF) variants, only those in genes with high intolerance to loss of function (pLI ≥ 0.9 or LOEUF ≤ 0.35) were considered likely to be causative^[^12^]^. Additionally, variants observed as heterozygous in unaffected family members were excluded from further consideration of pathogenicity.

Copy number variation (CNV) analysis was performed using the ExomeDepth (v1.1.15) and CODEX2 algorithms, with additional manual review of coverage plots. CNVs were filtered based on size (>1kb), quality metrics, and overlap with known pathogenic regions documented in the ClinGen and DECIPHER databases.

### Variant validation

Selected variants were validated using Sanger sequencing with custom-designed primers. PCR products were sequenced bidirectionally on an ABI 3730xl DNA Analyzer.

Because residual DNA was unavailable for several cases, Sanger validation was performed for 21 of 65 total variants (32.3%; see Supplemental Digital Content Table S1 at: https://links.lww.com/MS9/B338). For all validated variants, family segregation analysis was performed when possible. Variants observed as heterozygous in unaffected family members were classified as variants of uncertain significance (VUS) and excluded from pathogenicity assessment, in accordance with ACMG guidelines. The three retained likely pathogenic findings could not be Sanger-confirmed owing to insufficient residual DNA; this is acknowledged as a limitation of the study.

### Statistical analysis

Given the small sample size (*n* = 21) and the exploratory, retrospective design of this study, analyses were descriptive only. Continuous variables are summarized as mean ± SD, and categorical variables as n (%). No formal hypothesis testing or power calculation was performed; all findings should be interpreted as hypothesis-generating.

## Results

### Clinical characteristics

A total of 21 pediatric patients were included: 15 males and 6 females, with a mean age of 3.9 ± 3.0 years. Seizure types included febrile seizures in 9 patients (43%), non-febrile seizures in 6 patients (29%), and mixed-type seizures in 6 patients (29%). A family history of seizures was present in 13 of 21 patients (62%). Comorbidities included developmental delay (2/21, 10%), gastrointestinal disorders (5/21, 24%), and allergic conditions (2/21, 10%). Detailed clinical and demographic features are summarized in Table 1.

**Table 1:** Demographic and clinical characteristics of pediatric seizure patients (*N* = 21).

Characteristics	*n* (%) or Mean ± SD
Demographics	
Age (years)	3.9 ± 3.0
Age distribution	
< 1 year	2 (10)
1–3 years	7 (33)
4–6 years	7 (33)
7–10 years	5 (24)
Sex	
Male	15 (71)
Female	6 (29)
Clinical characteristics	
Seizure type	
Febrile seizures	9 (43)
Non-febrile seizures	6 (29)
Mixed type	6 (29)
Comorbidities	
Developmental delay	2 (10)
Gastrointestinal disorders	5 (24)
Allergic conditions	2 (10)
Neurological manifestations	1 (5)
None	14 (67)
Family history	
Positive	13 (62)
Negative	8 (38)
Affected family members	
First-degree relatives	4 (19)
Second-degree relatives	1 (5)
Multiple relatives	6 (29)
Positive (unspecified)	2 (10)

Note for Table 1: Comorbidity categories are not mutually exclusive.

### Sequencing findings and variant validation

WES identified 65 variants (63 sequence variants and 2 copy-number variants). Twenty-one variants (32.3%) were validated by Sanger sequencing. Three findings were retained as clinically significant likely pathogenic variants after ACMG/AMP-based interpretation and phenotype review; the remaining 62 variants were classified as VUS. The three retained clinically significant findings were not Sanger-confirmed due to insufficient residual DNA, which is acknowledged as a limitation (see Section 4).

### Clinically significant variants

Three clinically relevant, likely pathogenic findings were identified in three patients, corresponding to a diagnostic yield of 14.3% (Table 2): a GLI3 (exon 2: c.90_91insCAGATGTGAGC; p.Glu31Glnfs*3) frameshift mutation in patient P12 with febrile seizures, and two CNVs associated with established microdeletion/microduplication syndromes in P03 and P18. These three findings were either not observed in relevant population databases or involved recognized disease-associated genes or regions; however, none underwent Sanger validation because of insufficient DNA for confirmatory testing. The GLI3 c.90_91insCAGATGTGAGC frameshift variant may represent an expansion of the known phenotypic spectrum of GLI3 haploinsufficiency. This 1-year-old female patient (P12) presented with febrile seizures and a relevant family history, yet lacked the craniofacial and limb abnormalities characteristic of Greig cephalopolysyndactyly syndrome – the classical GLI3-associated disorder. The variant’s absence from genomic databases, combined with ACMG evidence (PVS1 + PM2_Supporting) and the gene’s high constraint profile (pLI = 1.0, LOEUF = 0.3), suggests that GLI3 haploinsufficiency may contribute to a seizure disorder in addition to its reported role in congenital malformation syndromes such as Greig cephalopolysyndactyly syndrome and Pallister–Hall syndrome. This finding suggests that highly constrained developmental genes may contribute to seizure phenotypes through mechanisms distinct from their classical syndromic presentations.

**Table 2:** Clinically relevant likely pathogenic findings in three patients.

Patient	Gene/genomic region	Variant	Protein change	Type	Zygosity	OMIM	Inheritance	Disease	Constraint assessment	Disease association	Population frequency (AF_eas)	Sanger validation	ACMG criteria Met	Final classification
P12	GLI3	Exon2:c.90_91insCAGATGTGAGC	p.Glu31Glnfs*3	Frameshift	Heterozygous	175 700	AD	Greig Cephalopolysyndactyly Syndrome	Highly constrained (pLI = 1, LOEUF = 0.3)	Greig Cephalopolysyndactyly Syndrome	Not observed in gnomAD East Asian population	Not validated	PVS1 + PM2_Supporting	Likely pathogenic
P03	16p13.12-16p13.11	1.70 Mb duplication	NA	CNV (1.70Mb dup)	Copy-number gain	–	NA	16p13.11 Microduplication Syndrome	CNV – constraint metrics not applicable	16p13.11 Microduplication Syndrome	Not applicable for CNV	Not validated	Known recurrent CNV region; phenotype overlap; interpreted with CNV-level evidence	Likely pathogenic
P18	Xp22.31	1.68 Mb deletion	NA	CNV (1.68Mb del)	Hemizygous	–	XLR	X-linked Ichthyosis	CNV – constraint metrics not applicable	X-linked Ichthyosis	Not applicable for CNV	Not validated	Known recurrent CNV region; phenotype overlap; interpreted with CNV-level evidence	Likely pathogenic

PVS1, very strong pathogenic; PM, moderate pathogenic; PP, supporting pathogenic; CNV, copy number variant; AD, autosomal dominant; XLR, X-linked recessive; NA, not applicable.

Population frequencies are based on gnomAD East Asian population data when applicable. For CNVs, population frequency is reported as not applicable.

### Variants of uncertain significance

**Figure 2.**
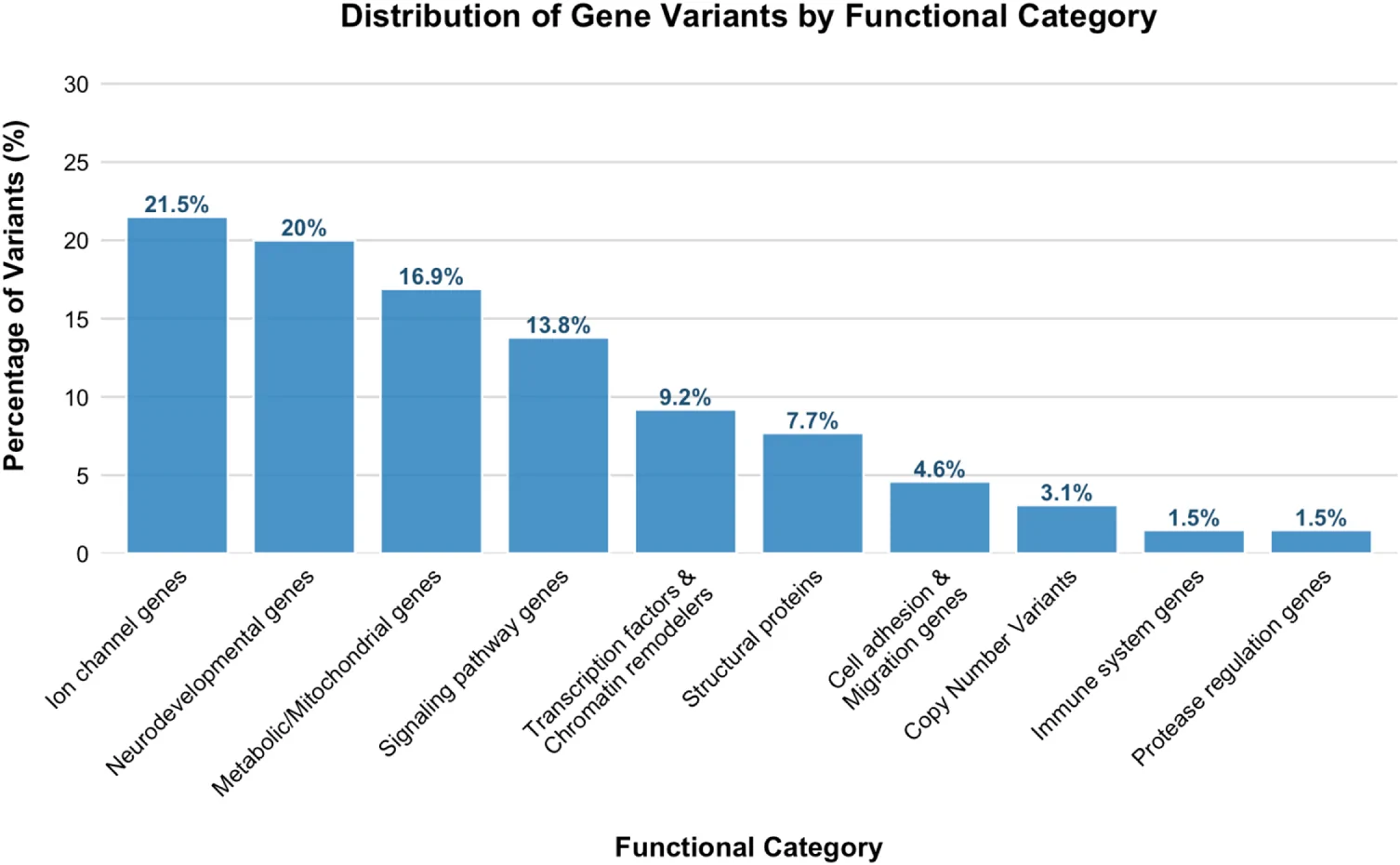
Functional Categorization of Genes with Variants. A bar chart shows the percentage distribution of variants across different functional categories, including ion channels, neurodevelopmental genes, and other categories.

Most variants (62/65, 95.4%) were classified as VUS according to the ACMG/AMP criteria and should not be considered diagnostic at this time. Functional analysis of the 65 genes harboring these variants revealed distinct functional categories (Fig. 2 and Table 3). Ion channel genes comprised the largest group (21.5%, 14 variants), including SCN1A, SCN8A, and CACNA1A. Neurodevelopmental genes represented 20.0% (13 variants), with genes such as RELN, MTOR, and NDE1 involved in brain development and synaptic function. Other major categories included metabolic/mitochondrial genes, signaling pathway genes, and transcription factors/chromatin remodelers.

**Table 3:** distribution of gene variants by functional category.

Category	No.	%	Genes identified (variant count)
Ion channel genes	14	21.5	SCN1A(2), SCN8A, CACNA1A, CACNA2D2, CACNB4, HCN2, CLCN2, GLRB, RYR1(2), FGF13, GRM7, EFHC1
Neurodevelopmental genes	13	20.0	RELN(2), MAB21L2, NEUROD2, MTOR, PRICKLE1, NDE1(2), FRMD4A(2), BRAT1, TBC1D24, CDK13
Metabolic/Mitochondrial genes	11	16.9	SDHB, ADA, PAH, ACADM(2), GPT2, GARS1, GAMT, HEXA, HDC, DIABLO
Signaling pathway genes	9	13.8	ADGRV1(3), ADCY5, GABBR2, MEFV(2), IRF5, PRKAG2
Transcription factors & Chromatin remodelers	6	9.2	KMT2C, ATRX, GLI3, TRRAP, BACH2, PAX1
Structural proteins	5	7.7	COL6A2, PIGO, TTN, LAMB1, PCDH15
Cell adhesion & Migration genes	3	4.6	FRRS1L, ADAM22, NCF1
Copy Number Variants (CNVs)	2	3.1	16p13.12-16p13.11 duplication, Xp22.31 deletion
Immune system genes	1	1.5	IGLL1
Protease regulation genes	1	1.5	SERPINI1

1. Total variants identified: 65 (63 sequence variants and 2 CNVs).

2. Percentages are calculated based on total number of variants.

3. Numbers in parentheses [e.g., (2), (3)] indicate multiple variants found in the same gene.

4. Genes may have multiple functions but are categorized by their primary role in seizure pathogenesis.

5. CNVs are included in total count and listed separately as structural genomic variants.

## Discussion

This study investigated the genetic landscape of pediatric seizure disorders in a Southeast Chinese cohort using WES. Three clinically significant, likely pathogenic findings were identified among 21 patients, corresponding to a provisional diagnostic yield of 14.3%. Published diagnostic yields in pediatric epilepsy sequencing studies range from approximately 20% to 50%, depending on study design, sequencing strategy (proband-only versus trio), phenotypic severity, variant classification stringency, and validation requirements^[^13–17^]^. Our lower provisional yield likely reflects the combination of small cohort size, phenotypic heterogeneity (including patients with febrile seizures not typically associated with monogenic causes), proband-only sequencing, strict ACMG/AMP interpretation, and limited Sanger confirmation. These factors are consistent with observations in similar retrospective single-center studies^[^9,10^]^.

The novel GLI3 frameshift variant (c.90_91insCAGATGTGAGC; p.Glu31Glnfs*3) is potentially important, as GLI3 is classically associated with Greig cephalopolysyndactyly syndrome and Pallister–Hall syndrome. Patient P12 presented with febrile seizures without syndromic features, and the variant was absent from population databases with strong constraint metrics (pLI = 1.0, LOEUF = 0.3). However, the absence of Sanger confirmation, incomplete segregation analysis, and lack of functional assays mean that this finding must be regarded as a candidate genotype–phenotype association rather than established causality. Future studies with functional validation are required before the phenotypic spectrum of GLI3 can be conclusively expanded to include seizure phenotypes. The discovery in a Southeast Chinese patient highlights the value of genetic studies in underrepresented populations for identifying population-specific variants and novel disease mechanisms.

While our approach successfully identified high-confidence variants, the substantial proportion of VUS warrants further discussion, particularly regarding variants with emerging epilepsy associations that may gain clinical significance through future research. A notable limitation of the present study is that 62 of the 65 identified variants (95.4%) were classified as VUS according to the ACMG/AMP criteria, reflecting a common challenge in rare disease genomics where insufficient population frequency data, functional evidence, or co-segregation data preclude definitive pathogenicity classification^[^11^]^. The high VUS rate is partly attributable to the broad functional heterogeneity of implicated genes. In ion channel genes such as SCN1A and SCN8A, VUS may confer either gain-of-function or loss-of-function effects with distinct clinical consequences, yet discriminating between these mechanisms from sequence data alone remains difficult^[^18^]^. Similarly, for metabolic genes such as SDHB, over 80% of missense variants cataloged in clinical databases are currently classified as VUS, highlighting the widespread interpretive gap in this gene class^[^19^]^. Neurodevelopmental genes, including RELN and NEUROD2, present analogous challenges due to their large coding sequences and variable penetrance. Future studies incorporating functional assays, larger case–control cohorts, and ClinGen expert panel curation will be essential to reclassify these VUS and improve the diagnostic yield and clinical utility of genomic findings in this patient population^[^20^]^.

Among these VUS, ADGRV1 represents a particularly interesting candidate gene for epilepsy. While ADGRV1 belongs to the adhesion GPCR family and its precise role in epileptogenesis requires further investigation, emerging evidence supports several potential mechanisms through which ADGRV1 variants may contribute to seizure susceptibility^[^21,22^]^. Additionally, ADGRV1 encodes VLGR1 (very large G protein-coupled receptor-1), which functions as a mechanosensor at focal adhesions^[^23^]^. Animal model studies provide compelling evidence demonstrating that VLGR1 domain loss causes audiogenic seizures in mice^[^24,25^]^. Clinically, ADGRV1 variants show significant overrepresentation in epilepsy cohorts, with frequencies ranging from 2.88% to 8.91% compared to controls, and are associated with favorable responses to antiepileptic drugs^[^24,26^]^. However, the ADGRV1 variants in this study remain VUS and should not be interpreted as diagnostic without segregation, case-control enrichment, or functional validation. We therefore present ADGRV1 only as an example of a candidate gene that may be reclassified as evidence accumulates.

Several limitations must be acknowledged: (1) small sample size (*n* = 21), limiting statistical power and generalizability; (2) retrospective design with variable clinical documentation; (3) heterogeneous phenotypic spectrum, including both febrile and non-febrile seizures; (4) proband-only sequencing without parental samples, limiting segregation analysis; (5) Sanger confirmation was performed for only 21 of 65 variants, and the three retained clinically significant findings were not confirmed by Sanger sequencing; and (6) absence of functional assays. Future studies should include larger multicenter cohorts, trio-based sequencing, standardized phenotyping protocols, and complete variant validation to improve diagnostic yield and confidence.

## Conclusions

WES identified clinically relevant, likely pathogenic variants in 3 of 21 pediatric patients with seizure disorders from Southeast China. The diagnostic yield of 14.3% should be interpreted cautiously, given the small cohort and limited validation. The novel GLI3 frameshift variant may represent a potential expansion of GLI3-associated phenotypes, but further segregation and functional studies are required before drawing definitive conclusions. The high VUS rate underscores the importance of larger population-specific genomic datasets, standardized phenotyping, and functional validation to improve the interpretation of genetic findings in pediatric seizure disorders.

## Data Availability

The raw sequence data reported in this paper have been deposited in the Genome Sequence Archive at the National Genomics Data Center, China National Center for Bioinformation/Beijing Institute of Genomics, Chinese Academy of Sciences, under the accession number GSA-Human: HRA009802. The data are accessible upon request through the Genome Sequence Archive.

## Research registration unique identifying number

Not applicable. This retrospective, non-interventional observational study used existing clinical records and WES data. No participants were prospectively assigned to an intervention, and no intervention was evaluated; therefore, study registration was not required.

## Assistance with the study

We thank Ying Zhou and Jialin Zhao from the Medical Statistical Consultancy Services Program, National Institute for Data Science in Health and Medicine, School of Medicine, Xiamen University, for statistical support.
